# A study of patient attitudes towards decentralisation of HIV care in an urban clinic in South Africa

**DOI:** 10.1186/1472-6963-11-205

**Published:** 2011-08-26

**Authors:** Rachel Mukora, Salome Charalambous, Maysoon Dahab, Robin Hamilton, Alan Karstaedt

**Affiliations:** 1 Aurum Institute, Johannesburg, South Africa; 2 Adult HIV Clinic, Chris Hani Baragwanath Hospital and University of the Witwatersrand, Johannesburg, South Africa

**Keywords:** decentralization, down-referral, South Africa, ART, level of care

## Abstract

**Background:**

In South Africa, limited human resources are a major constraint to achieving universal antiretroviral therapy (ART) coverage. Many of the public-sector HIV clinics operating within tertiary facilities, that were the first to provide ART in the country, have reached maximum patient capacity. Decentralization or "down-referral" (wherein ART patients deemed stable on therapy are referred to their closest Primary Health Clinics (PHCs) for treatment follow-up) is being used as a possible alternative of ART delivery care. This cross-sectional qualitative study investigates attitudes towards down-referral of ART delivery care among patients currently receiving care in a centralized tertiary HIV clinic.

**Methods:**

Ten focus group discussions (FGDs) with 76 participants were conducted in early 2008 amongst ART patients initiated and receiving care for more than 3 months in the tertiary HIV clinic study site. Eligible individuals were invited to participate in FGDs involving 6-9 participants, and lasting approximately 1-2 hours. A trained moderator used a discussion topic guide to investigate the main issues of interest including: advantages and disadvantages of down-referral, potential motivating factors and challenges of down-referral, assistance needs from the transferring clinic as well as from PHCs.

**Results:**

Advantages include closeness to patients' homes, transport and time savings. However, patients favour a centralized service for the following reasons: less stigma, patients established relationship with the centralized clinic, and availability of ancillary services. Most FGDs felt that for down-referral to occur there needed to be training of nurses in patient-provider communication.

**Conclusion:**

Despite acknowledging the down-referral advantages of close proximity and lower transport costs, many participants expressed concerns about lack of trained HIV clinical staff, negative patient interactions with nurses, limited confidentiality and stigma. There was consensus that training of nurses and improved health systems at the local clinics were needed if successful down-referral was to take place.

## Background

In order to rapidly increase access to antiretroviral treatment (ART) to all those who require it in sub-Saharan Africa, developing countries need to devise strategies to increase provision of ART within the constraints of limited resources [1]. In South Africa, limited human resources to treat HIV-infected patients is one of the main constraints to achieving universal ART coverage [1,2] and will remain an obstacle in ART treatment. Staffing problems include emigration of health workers, limited training opportunities, illness among health workers and increased demand for treatment [3-5]. Task-shifting (the delegation of medical and health service duties from higher to lower levels or new levels of trained staff) and decentralization of HIV services have been proposed as solutions to the staffing shortage [6]. Other advantages cited for task shifting include improved workforce skills mix, enhancing the role of the community and cost advantages. Challenges sited previously include addressing resistance to change and sustaining motivation and performance [6].

By 2007, some of the first clinics to offer ART in Gauteng Province had reached capacity owing to limited physical and human resources, and so the initiation of new patients had slowed down. In response, the Gauteng Department of Health (DOH) introduced the policy of down-referral. According to this policy, ART patients started treatment at HIV clinics operating within secondary or tertiary facilities, once stabilized on treatment, could be referred to primary health centers (PHCs) for continued care and treatment.

The Chris Hani Baragwanath Hospital (CHBH) is the only public-sector hospital in the large township of Soweto, within which in 2004 the adult HIV clinic was the first accredited site for ART provision in the township. In 2007, the adult HIV clinic began exploring the down-referral policy. However, with the lack of published studies on down-referral of patients with HIV or other chronic diseases in the current medical literature, information on how patients may perceive and ultimately respond to treatment once down-referred were greatly lacking. Given this dearth of information we undertook this study to determine patients' perception of the benefits as well as obstacles of down-referral from the adult HIV clinic to PHC in Soweto. This is in order to better inform how down-referral can best be implemented to ensure good treatment outcomes at both the individuals and population levels.

## Methods

We held focus group discussions (FGDs) with patients to investigate patient attitudes towards down-referral of HIV care from CHBH to PHCs in Soweto. Adult patients who had been on antiretroviral therapy for 3 months or more and who were willing to speak openly in a focus group were invited to participate in the study.

### Study Site

CHBH is a 2 700 bed public hospital. The CHBH Adult HIV Clinic was opened in 1989 and by mid-2010 had more than 4 500 patients on ART. The initiating criteria and treatment regimens follow national guidelines and treatment is offered free of charge [7]. The clinic is staffed by infectious diseases specialists, a registrar in internal medicine, medical officers, professional nurses, counsellors and data capturers. Laboratory testing, a pharmacy, radiology and other specialist services are easily accessible within walking distance and within the same hospital complex. Patients seen at this clinic are largely female, have high proportions of unemployment and have an average of 11 years of education [8]. The HIV prevalence appears to be consistent over the years and the rate stood at around 30% amongst the antenatal attendees in 2007 [9].

### Study Procedures

A research nurse introduced the study to ART patients in the waiting room of the Adult HIV Clinic. Patients were requested to attend an education session if they were interested in a group discussion about the clinic and the concept of downreferral. Once all assembled, participation in the FGD was explained in detail and those attending could volunteer to be in the study. Most patients who attended the education session either agreed to participate immediately or stated that they would return on another day if they didn't have enough time. No patients were turned away. If an individual agreed to participate s/he would be given information about the time and location of their assigned focus group. Written informed consent was obtained from all participants. A trained moderator, who was an independent research staff member not involved in patient care, led each FGD in local languages (Sesotho, Zulu or Setswana) and using a moderator guide.

The guide was developed based on FGD principles outlined by Green et al. [10] and included the following topics:

1. Do participants believe down-referral to be a good or bad thing?

2. What are motivating factors for down-referral?

3. What are the challenges of down-referral?

4. What help would patients need if they were down-referred?

5. What access to the initiating clinic would patients want?

### Data handling and analysis

Each Discussion was tape recorded and the moderator's assistant also took detailed notes of the discussion, including spontaneous conversation among participants prior to and following the group discussion. Discussions were transcribed verbatim and translated into English (if they weren't done in English). Transcripts was analysed using thematic content analysis in which a coding scheme was used to identify the most common or recurrent themes in the data [11]. The first author abstracted the information from the FGDs and identified recurring themes. These themes were checked for validity by the second author and after discussion, consensus was reached on the themes. The themes were then categorized as being either an advantage or a disadvantage of down-referral.

### Ethical considerations

The study team obtained approval for the study from the Human Research Ethics Committee of the University of the Witwatersrand, and the Ethics Committee of the London School of Hygiene and Tropical Medicine, UK.

The research staff obtained written informed consent from participants. The participant information sheet and consent form were available in the most commonly used local languages (Sesotho, Xhosa, Zulu and Setswana). Participants who were unable to read had the study information sheet and consent form read to them in the language of their choice by a trained member of the study staff who was fluent in the language spoken by the participant. The consent process was witnessed by an individual fluent in the language in which the consent was obtained.

## Results

Ten FGDs were held between 15 February and 7 March 2008, with 6-9 participants in each focus group (labeled A-J). There were 76 participants: 27 (36%) males and 49 (65%) females. The most common home languages were Sesotho, Zulu and Setswana (Table 1).

**Table 1:** Participant demographics and attitude to down-referral by focus group

Focus group	Number of participants	Gender		Home languages	Groups in favour of down-referral	Groups opposed to down-referral
		**Male**	**Female**			

**A**	**7**	**1**	**6**	Sesotho, Zulu	**3 (43%)**	**4 (57%)**

**B**	**8**	**1**	**7**	Sesotho, Setswana, Zulu	**0 (0%)**	**8 (100%)**

**C**	**8**	**6**	**2**	Sesotho, Zulu	**4 (50%)**	**4 (50%)**

**D**	**8**	**3**	**5**	Sesotho, Setswana, Zulu	**4 (50%)**	**4 (50%)**

**E**	**8**	**5**	**3**	Sesotho, Zulu, English	**1 (13%)**	**7 (88%)**

**F**	**9**	**4**	**5**		**4 (44%)**	**5 (56%)**

**G**	**8**	**3**	**5**	Sesotho, Setswana, Zulu	**4 (50%)**	**4 (50%)**

**H**	**6**	**0**	**6**	Sesotho, Setswana, Zulu	**2 (33%)**	**4 (67%)**

**I**	**8**	**3**	**5**	Sesotho, Setswana, Zulu	**4 (50%)**	**4 (50%)**

**J**	**6**	**1**	**5**	Sesotho, Setswana, Zulu	**2(33%)**	**4(67%)**

**Total**	**76**	**27**	**49**		**28 (37%)**	**48 (63%)**

Six out of ten groups had a majority that voted against down-referral, whilst in four groups participants seemed equally divided regarding down-referral. No group had a majority that voted in favor of decentralization. Table 2 indicates the number of groups that mentioned each theme. The following section is divided into two sections: perceived advantages and disadvantages of down referral.

**Table 2:** Perceived advantages and disadvantages of down-referral as mentioned by focus groups

Advantages	Number of focus groups	Disadvantages	Number of focus groups
Proximity to clinics	8	Stigma	10

Transport savings for patients	8	Poor service at clinics	9

Necessity, owing to patient load at tertiary hospital	7	Inadequate drug supply	9

Time savings for patients	4	Preference for access to doctors	9

Respectful/helpful nurses at clinics	3	Lack of professionalism of nurses	7

		Convenience of hospital	6

		Fear of mistreatment at clinics	6

		Familiarity with health-care workers at tertiary hospital	5

		No time saving attending clinic	4

		No transport saving attending clinic	4

### Advantages of down-referral

#### Close to patients' homes

When asked about the advantages of down-referral, members of most of the focus groups mentioned that down-referral to local clinics would allow them to be able to access health-care closer to their homes. This was especially important in times when the patients were too ill to travel long distances to the hospital:

"... at times you are unable to walk, so if you are near the clinic, you can try to walk to that clinic, sometimes you are at home with no one to accompany you to [the hospital], at times you do not even have money for transport, so it will be better to go to the nearest clinic." (Focus group B)

"... it will help those people, who travel long distances to come to [the hospital], it will be better as they can go to their nearest clinics to get treatment and medication..." (Focus group E)

#### Transport savings

Participants in a majority of groups said that attending local clinics was advantageous in that it would help save on transport costs which many struggled to afford:

"Many people are not working, so it is difficult to come here [to the hospital] because of money problems... down-referral will be very good for economic reasons as they can just walk to the clinic." (Focus group C)

However, a few FGDs felt that the transport costs to their PHC and to the hospital were equivalent and so there were no transport savings gained by attending the PHC. According to a participant in one of the FGDs:

"From home to [PHC] Clinic or from home to [the hospital] I pay the same amount of money round trip, so it does not save me any money either way." (Focus group A)

#### Time saving

Most of the FGDs thought that time could be saved by attending local clinics, which were perceived as less busy. It would also mean spending less time away from work for those patients who are employed:

"Down-referral is going to help with the queues as there are so many of us with HIV/AIDS... it is going to help patients who come from far who spend a lot of time to the clinic." (Focus group F)

In contrast, a few groups felt that there was no time saved by attending local clinics, which were perceived as already overcrowded:

"The bad side of the clinic is that right now they are overloaded... that is a real problem. So if now they have to add HIV-positive patients, they will tell us about the time, at 12h00, they cut the number of patients to be seen." (Focus group C)

#### Altruism

Despite their preference for being seen at CHBH, down-referral was seen as a necessity by a majority of the respondents, since this measure would allow new HIV-positive patients to be seen at the CHBH clinic:

"... I think it is right to be down- referred as we are now feeling better. It was sad when we were ill with no one to treat us; we must also now think of other ill patients." (Focus group D)

### Disadvantages of down-referral

#### Fewer services at the local clinics

Some of the focus group members said it was more convenient to attend the hospital with a range of health services within easy reach, as it was then easier to be referred for other forms of care:

"Here at [the hospital] everything is nearer... they can refer me as quickly as possible. I had instances when I was ill, was mentally disturbed and needed a psychologist, they referred me quickly, unlike if I am at the local clinic, it will take a long time to come here." (Focus group G)

#### Loss of established relationships with hospital health-care workers

Half the respondents felt that down-referral was disadvantageous as they would lose their long-established relationships with health-care workers at the hospital:

"The bad aspect will be to lose the relationship we have with our doctors...She [the doctor] is like a mother here in the hospital. Now we have to start afresh with new people and we do not know what to expect." (Focus group H)

#### Loss of doctor-based care

Participants believed that the doctors at the adult HIV clinic had had better training and awareness of HIV/AIDS than nurses, and thus doctors were perceived as taking better care of patients. The respondents worried that they would need to seek their doctor's care after being down-referred:

"I have realized that here at [the hospital] all the doctors are specialists in HIV/AIDS, so if we go to the clinics... will those sisters be able to treat such opportunistic infection, or will we be faced with a problem of coming back to [the hospital] after being down-referred?" (Focus group C)

Thus participants in most groups feared losing direct access to doctors if down-referred to PHC:

"The clinic nurses are not good, that is why we all come here as... at least here [at the hospital] the doctor will enquire after your health. In the [local] clinic you cannot even ask [nurses] the function of the tablets they give you." (Focus group E)

Therefore, some believed down-referral could be acceptable but only for treatment pick up, while routine quarterly care should still provided by doctors at the adult HIV clinic:

"I can go there [local clinic] to fetch my medication, but would like to come here [to the hospital] every 3 months to see my doctor". (Focus group H)

#### Mistreatment by PHC nurses

Many perceived PHC nurses as having negative attitudes towards patients. The respondents complained that the PHC nurses even went as far as revealing the patients' status publicly:

"... the nurses in the clinics have bad manners, the sisters in the clinic tell everybody about your status that is why we are afraid to go to the local clinics. Here at [the hospital], we do not get problem from the nurses." (Focus group F)

Hence, a clear majority of the FGDs expressed fear that they would be mistreated by nursing staff if down-referred to PHCs:

"From the clinics it's not the same as here at [hospital]. When you come here [to the hospital] you feel welcome, the nurses greet you and enquire after your health, they have that smile, but at the clinic it's only,' we do not have such a thing, go somewhere else', they cannot talk to us." (Focus group B)

#### Stigma at local clinics

Participants also expressed concerns about stigma and loss of confidentiality where they were more likely to meet neighbors, friends, or other people they know but do not wish to share their HIV status with:

"Firstly in our area where I stay, we know each other, so at the end of the day you find people pointing fingers at you in the clinic talking about you and all that and you end up being afraid to go for your treatment." (Focus group A)

"I know of two people who take ART at [the clinic] they say it is very full and when you go to 'that side' it means you have something, even if you are open about your status at times you feel bad when people point fingers at you that you have something else since you go to that 'other department' in the clinic for 'those people', as if we are not human beings like other people. I think things are better here at [the hospital] and I would not like to be referred anywhere." (Focus group B)

#### Poor service at clinics

In summary, almost all the FGDs agreed that the hospital provided a good service while the service at the local clinics was poor:

"It's OK here at [the hospital] because we get all the treatment we need... We are not well treated at the clinic, we are harassed and not regarded as human beings and end up not getting the correct medication..." (Focus group B)

### Suggested solutions to potential problems with down-referral to phcs

Several suggestions were put forward by the participants in order to improve services at the PHC level:

#### Improve clinic health systems

Nearly all of the focus groups felt that if down-referral was to take place then systems would need to be better organized within the clinics:

"... we need to go there when we are sure that all the systems are in place." (Focus group C)

"At the clinics they could allocate clinic days according to the zones we come from, or say some days will be doctor's day and other days for just collecting pills, because there are no doctors in the local clinics. Training of the nurses is very important and medication should be available at all times" (Focus group B)

#### Provide training to staff

Most FGDs believed that training should be mandatory for nurses at local clinics in both HIV/AIDS and in interpersonal skills:

"Some of the clinics provide a good service but some of the clinics... no, no they go for tea for 2 hours and you become so hungry that you feel like fainting, so if they can just sort things out first in the clinics and teach them how to handle patients with our conditions and have good interpersonal relationships with other people, then I think we will ask to be down referred" (Focus group A)

"There should be 100% recruitment of nurse who will specialize in this HIV/AIDS and get training. We need a team that will specialize in this disease." (Focus group C)

#### Patient tolerance of health workers

Less than half of the focus groups thought that HIV-positive patients should be more tolerant of the poor behavior of nurses at local clinics:

"About the nurses' rudeness, I think the nurses should be informed how to treat a patient, the patient must also have the patience, understand the nurses and like I, I do not have any problems with my nurses in my local clinic because of the people's person I am, because whenever they see my file they will always assist me." (Focus group A)

#### Improve drug supply

Poor drug supply, which was cited as an issue in most of the groups, would also have to be improved for the patients to regain their confidence in attending local clinics:

"Will we always get the ARV's as in the clinics they always have no Panado or cough mixture, how sure are we that we will always get our ARVs?" (Focus group A)

## Discussion

In this study, concerns about down-referral seemed to outweigh perceived advantages. Patients favoured a centralised service for the following reasons: easier availability of ancillary services, a pre-existing relationship with the centralized clinic, friendlier nurses and less stigma. Stigma remains an important issue for HIV-positive people, and patients perceived a hospital service to provide greater anonymity and less stigma. These findings are in keeping with another study in Johannesburg that found that patients opted to avoid clinics in their local areas, and preferred to travel to more distant facilities so as to conceal their HIV status from community members [12]. The fear of stigma may also impact negatively on patients' adherence to treatment [13,14].

Decentralisation of services has been shown to be successful in a number of other African countries with regards to TB treatment and care [15,16]. For this reason, task sharing has been recommended in HIV programmes in order to improve the access of HIV-positive people to ART and to facilitate retention of patients on ART programmes [6,11].

Decentralisation of services, with good retention of patients and acceptable virological outcomes has been described in South Africa in various districts [17,13]. In addition, there is evidence that services provided closer to the patient's home result in improved retention compared to services provided centrally [12,14]. A study in Malawi comparing patients started on treatment at a central hospital and at peripheral health centers found similar attrition rates at different types of facility, but increased losses to follow-up at the central hospital and higher mortality at the peripheral health center. The increased mortality at the peripheral health center was attributed to a better ascertainment of the causes of death rather than a real increase in deaths [18]. In South Africa, a comparison of outcomes in patients on ART showed improved virological response and retention in primary health centres compared to district hospitals and regional hospitals [13]. There have also been reports of reduced effectiveness of peripheral programmes such as in a study in China that showed that village clinics and township health centres were less likely to achieve viral suppression compared to county-level hospitals [14].

Familiarity with health service providers was raised frequently by focus group members as an important aspect of good care. This may have also been influenced by a policy within the CHBH clinic which encouraged patients to see the same providers at each visit. The patient-provider relationship has long been identified as an important element of any health service. Good provider-patient relationships improve patients' adherence to ART [19-24].

Another concern raised by focus group members about the decentralization of care was that peripheral clinics were already overcrowded with patients with other chronic conditions. Other difficulties with down-referral included perceived lack of training of nurses and thus patients' preference to be seen by doctors who were perceived as more experienced. Nurses' lack of professionalism and fear of mistreatment was seen as contributing to patients' reservations about down-referral. When planning decentralization, efforts need to be taken to ensure clinics fulfill certain criteria such as the availability of trained nurses and the safe storage of drugs, prior to transfer of patients, as has been done in Malawi [25]. Formal training of the nurses in HIV care and in effective patient-provider communication would be invaluable. Adequate support and monitoring of clinic staff may also contribute to decentralization being successful [26].

The main advantage of down-referral expressed by focus group participants was that local clinics were closer to patients' homes and were usually easier to reach, especially when participants were ill. A qualitative study using in-depth interviews with patients who had been lost to follow-up at the same hospital clinic found that one of the main reasons reported for attrition was not having funds to pay for transport to the clinic [27].

There are a number of potential limitations to this study. Patients who were interviewed may not be representative of the clinic population, as they had all been retained in care. As the FGDs were run in the CHBH clinic patients may have been less inclined to express any negative feelings about their current care providers at the hospital. Patients at the central hospital clinic may not have been representative of all patients needing to access HIV care. Patients in the FGD were only familiar with the hospital clinic setting for HIV care, and so would naturally have been more skeptical about another setting and more favourably disposed to centralized care. A similar study run at one of the initiating peripheral clinics might show different results, with high levels of satisfaction for peripheral clinic care.

## Conclusion

The study emphasizes the importance of patient attitudes to health interventions and highlights the importance of training and monitoring of health workers involved in the care of HIV-positive patients. There is a tension between the right of the patient to choose a provider and a site of care, and the need of the health service to down-refer patients to peripheral clinics,. Downreferral of patients need to acknowledge and take into consideration patients' perceptions of health services and their health-care preferences.

## Abbreviations

ART: Antiretroviral therapy/treatment; CHBH: Chris Hani Baragwanath Hospital; DOH: Department of Health; FGDs: Focus Group Discussions, PHCs Primary Health Clinics.

## Competing interests

The authors declare that they have no competing interests.

## Authors' contributions

RM: initial analysis of data and drafting of the manuscript.SC: support with protocol development, support with data collection, second review of analysis and manuscript development; MD: wrote study protocol, advised on data collection and reviewed the manuscript. RH: analysis and manuscript development AK: protocol development, data collection, analysis and manuscript development. All authors read and approved the final manuscript.

## Pre-publication history

The pre-publication history for this paper can be accessed here:

http://www.biomedcentral.com/1472-6963/11/205/prepub
